# CHCHD2 promotes hepatocellular carcinoma and indicates poor prognosis of hepatocellular carcinoma patients

**DOI:** 10.7150/jca.31158

**Published:** 2019-11-01

**Authors:** Yang Yao, Jie Su, Lei Zhao, Rong Li, Kaige Liu, Shengyu Wang

**Affiliations:** 1 Department of Central laboratory, The First Affiliated Hospital, Xi'an Medical University, Xi'an, Shaanxi 710077, PR China; 2 Department of Molecular Physiology and Biophysics, Holden Comprehensive Cancer Center, University of Iowa Carver College of Medicine, Iowa City, IA 52242, USA.; 3 Department of Ophthalmology, The First Affiliated Hospital of Xi'an Medical University, Xi'an, Shaanxi 710077, PR China; 4 Department of Gastroenterology, the First Affiliated Hospital, Xi'an Medical University, Xi'an, Shaanxi 710077, PR China.; 5 Department of Pulmonary and Critical Care Medicine, The First Affiliated Hospital of Xi'an Medical University, Xi'an, Shaanxi, 710077, China

**Keywords:** Hepatocellular carcinoma, CHCHD2, CD105, biomarker

## Abstract

The coiled-coil-helix-coiled-coil-helix domain containing 2 (CHCHD2) is overexpressed in several types of cancer. This study aimed to investigate the role of CHCHD2 in hepatocellular carcinoma (HCC). The expression of CHCHD2 in HCC and non-tumorous tissues was detected by immunohistochemistry and Western blot analysis, and the correlation between CHCHD2 expression and clinicopathological features of HCC was analyzed. Furthermore, the proliferation, apoptosis and migration of HepG2 cells with CHCHD2 knockdown were examined. We found that CHCHD2 was upregulated in HCC tissues, and high CHCHD2 expression was associated with poor differentiation, lymph node metastasis, local tissue invasion, high TNM grade of HCC and poor patient survival. Depletion of CHCHD2 led to significantly reduced cell proliferation, increased apoptosis and diminished migratory capacity in HepG2 cells. In addition, HCC tissues had high expression of CD105, a microvessel marker, and HepG2 cells depleted of CHCHD2 had low CD105 expression. In conclusion, CHCHD2 may play an oncogenic role in HCC via promoting tumor cell growth and migration while preventing apoptosis. CHCHD2 is a potential biomarker for poor outcome of HCC patients.

## Introduction

Hepatocellular carcinoma (HCC) is the most common aggressive hepatic cancer and ranks the fifth most prevalent malignancy worldwide 1,2. HCC is also one of the most commonly diagnosed cancers in China, accounting for 50% of liver cancer cases and death 2. The mortality of HCC is mainly due to spread via the lymphatic and blood system as a result of late-stage detection 3,4. Therefore, it is urgent to further understand the underlying mechanisms of HCC development and progression and identify novel targets for developing therapeutic strategy of HCC.

Coiled-coil-helix-coiled-coil-helix domain-containing protein 2 (CHCHD2) is a mitochondrial protein encoded by the CHCHD2 gene located on human chromosome 7P11.2 5, 6. CHCHD2 contains a conserved C-terminal CHCH domain and an N-terminal mitochondrion localization sequence 7. CHCHD2 overexpression has been found in a variety of cancers 8. However, the role of CHCHD2 in HCC is largely unclear.

In this study, we examined the expression of CHCHD2 in HCC and investigated the correlation between CHCHD2 expression and the clinicopathological features of HCC patients. In addition, we explored the function of CHCHD2 in HCC cells. We found that CHCHD2 was upregulated in HCC tissues and higher CHCHD2 protein level was significantly associated with poor differentiation, lymph node metastasis, local tissue invasion, high TNM grade, angiogenesis and poor prognosis in HCC patients. Moreover, depletion of CHCHD2 led to significantly reduced proliferation and invasion and angiogenesis and increased apoptosis in HepG_2_ cells.

## Materials and methods

### Patient specimens

A total of 144 HCC patients were enrolled in this study who received surgery at the first affiliated hospital of Xi'an Medical University from January 2005 to July 2012. HCC patients were diagnosed by histological examination of surgical specimens. The clinicopathological characteristics including age, gender, and tumor size and differentiation were collected by reviewing their medical records and summarized in Table 1. No patients received pre-operative radiotherapy or chemotherapy. All patients had a follow-up record for at least five years. This study has been approved by the Institute Research Medical Ethics Committees of the first affiliated hospital of Xi'an Medical University.

### Cell culture and transfection

HCC cell line HepG2 was obtained from the cell bank of the First Affiliated Hospital of Xi'an Medical University, and cultured in RIPM 1640 medium supplemented with 10% fetal bovine serum (Giboco BRL, USA) at 37°C in humidified atmosphere of 5% CO_2_. Transfection was performed using Lipofectamine 2000 (Life Technologies, USA). HepG_2_ cells were transfected with either siRNA targeting CHCHD2 (siCHCHD2) (Ruibo, Guangzhou, China) or non-targeting control siRNAs (NC). The targeting sequences of CHCHD2 siRNA oligos were as follows: 5'-GGTACCCTTTGGGGGGAACAGGTGGT-3', 5'-GGTACCGTTGACCGCGAAGGACGAG-3'.

### Immunohistochemical staining

Tissues blocks were dewaxed, hydrated and washed in PBS, and then immersed in 3% H_2_O_2_ for 20 min to inhibit endogenous peroxidase activity. After blocking, the sections were incubated for 2 h at room temperature with polyclonal rabbit anti-human CHCHD2 antibody (1:600; Santa Cruz, CA, USA) and CD105 antibody (1:100; Abcam, USA). After washes for three times, sections were incubated with peroxidase conjugated secondary antibody (1:1000, Bio-Rad, CA, USA) for 1 h at room temperature. After three additional washes, immunoreactivity was detected with diaminobenzidine (DAB) at room temperature and examined under a fluorescence microscope (Axioplan2; Carl Zeiss; Jena, Germany).

### Western blot analysis

Liver tissues or cells were homogenized in RIPA buffer (Beyotime Institute of Biotechnology, Haimen, China) and the protein concentrations were quantified by BCA assay (Santa Cruz Biotechnology, Santa Cruz, CA, USA) according to the manufacturer's instruction. A total of 40 µg lysates were separated by SDS-PAGE and transferred to PVDF membrane, which was then blocked with 5% non-fat dry milk for 1 h. The membranes were incubated with CHCHD2 antibody (1:400, Santa Cruz Biotechnology, Santa Cruz, CA, USA) and CD105 antibody (1:200, Santa Cruz Biotechnology, Santa Cruz, CA, USA) at 4°C overnight, followed by incubation with horse radish peroxidase conjugated secondary antibody (1:600, Santa Cruz Biotechnology, Santa Cruz, CA, USA) at room temperature for 1 h. ECL detection reagent (Santa Cruz Biotechnology, Santa Cruz, CA, USA) was used to detect the immunereactive bands. All bands were imaged and analyzed by densitometry scanning (Chemilmager IM5500, Alpha Innotech, USA).

### MTT assay

The cells were seeded onto 96-well plates at a density of 1×10^4^ cells/well and cell viability was measured in 24, 48 and 72 h. At each time point, 20 µl MTT reagent (5 mg/ml, Sigma-Aldrich, USA) was added to each well and allowed to incubate for 4 h. Then 150 µl DMSO was added into each well and the plates were incubated overnight. Absorbance at 490 nm was measured by using a microplate spectrophotometer (Epoch2, BioTek, USA).

### TUNEL assay

Apoptotic cells were detected by TUNEL assay using In situ Cell Death Detection kit (Roch, Pleasanto, CA, USA) according to the manufacturer's protocol. The number of apoptotic cells was counted in five randomly selected fields and expressed as a percentage of the total number of cells in the same field (apoptosis index).

### Wound healing assay

Cells transfected with siCHCHD2 or NC siNRA were reseeded in a 6-well culture plate at 5×10^5^ cells/well and incubated overnight. Then a line was scraped with a 10 μl pipette tip, and the cells were washed 3 times with PBS to remove detached cells. Space filling by cell migration was recorded at 0 and 24 h and analyzed by using Image Pro Plus 6.0 software (Media Cybernetics, Inc., USA).

### Statistical analysis

Data analyses were performed with SPSS 16.0 statistical software (SPSS, Chicago, IL, USA). All date were presented as mean ± standard deviation (SD). The differences between two groups were compared with χ^2^ test. The means of three or more groups were compared with one-way analysis of variance (ANOVA) followed by the Tukey post-hoc test. A *p*-value < 0.05 was considered statistically significant.

## Results

### CHCHD2 is upregulated in HCC tissues

**Figure 1:**
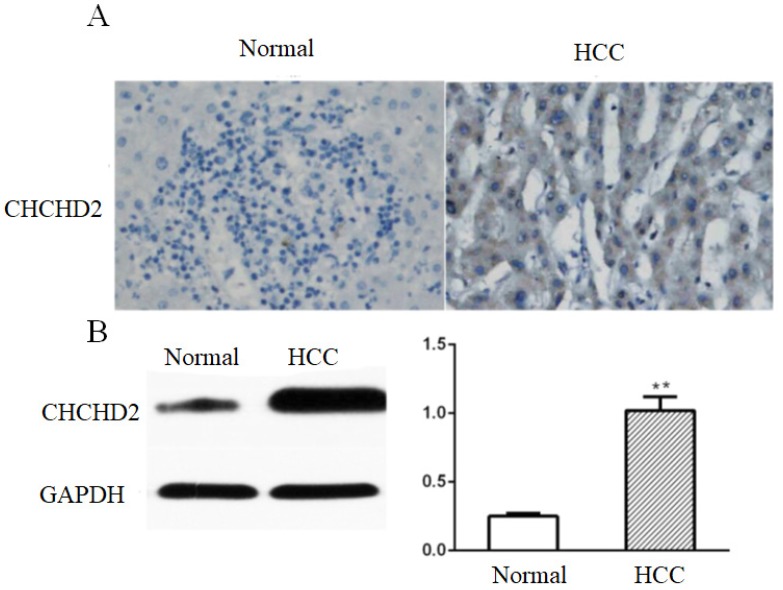
CHCHD2 is upregulated in HCC tissues. (A) Representative IHC images of CHCHD2 expression in the array that contained HCC and normal liver tissues. Original magnification: 400 x. (B) Western blot analysis of CHCHD2 level in HCC tissues and surrounding normal tissues. Data were presented as means±SD (n=3). ^* *^ *P*<0.01.

We assessed CHCHD2 expression in 144 HCC tissues and matched surrounding non-tumorous tissues by immunohistochemistry (IHC). The results showed that CHCHD2 was mainly expressed in the cytoplasm, and IHC score of CHCHD2 was significantly higher in HCC tissues compared to surrounding non-tumorous tissues (3.1 versus 1.0,* p*<0.05, Fig.1A). Western blot analysis confirmed that CHCHD2 expression was significantly higher in HCC tissues compared to normal liver tissues (*p*<0.05, Fig. 1B).

### Correlation between CHCHD2 expression and clinicopathological characteristics of HCC

The correlation between CHCHD2 expression and clinicopathological parameters of HCC patients was summarized in Table 1. The results indicated that high expression of CHCHD2 was significantly associated with poor differentiation, lymph node metastasis and high TNM stage, but not associated with the age, gender, and tumor size.

### High CHCHD2 expression is correlated to poor patient survival

**Figure 2:**
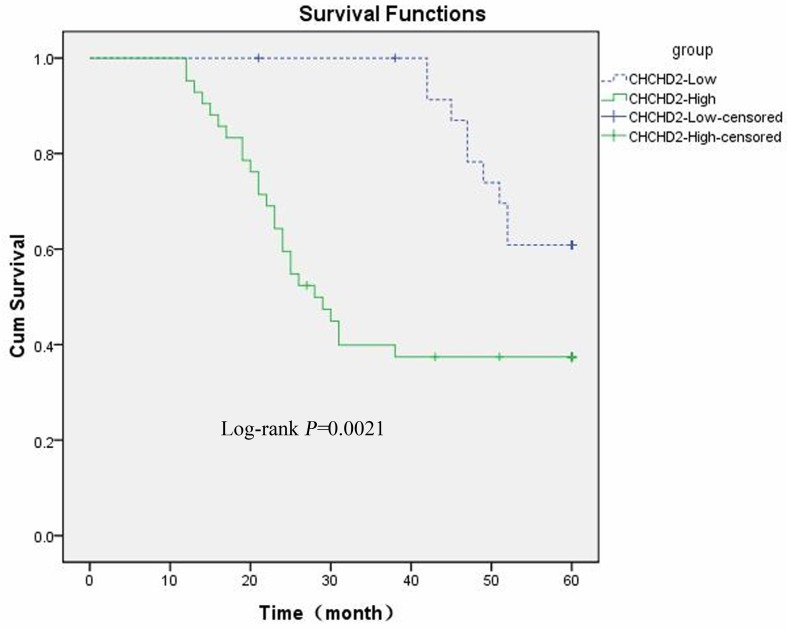
High CHCHD2 expression level is correlated to poor patient survival. The five-year survival rate was analyzed by Kaplan-Meier method. The Log-rank test was used to compare the growth curve between patients with low and high expression level of CHCHD2.

The prognostic value of CHCHD2 for HCC patients was investigated by using Kaplan-Meier survival curve and Log-rank test. As shown in Figure 2, the five-year overall survival of patients with high and low CHCHD2 expression was 43.3% and 81.1% (Log-rank *p*<0.05), respectively, suggesting that CHCHD2 level is negatively associated with the outcome of HCC patients.

### CHCHD2 knockdown decreased proliferation and enhanced apoptosis of HCC cells

**Figure 3:**
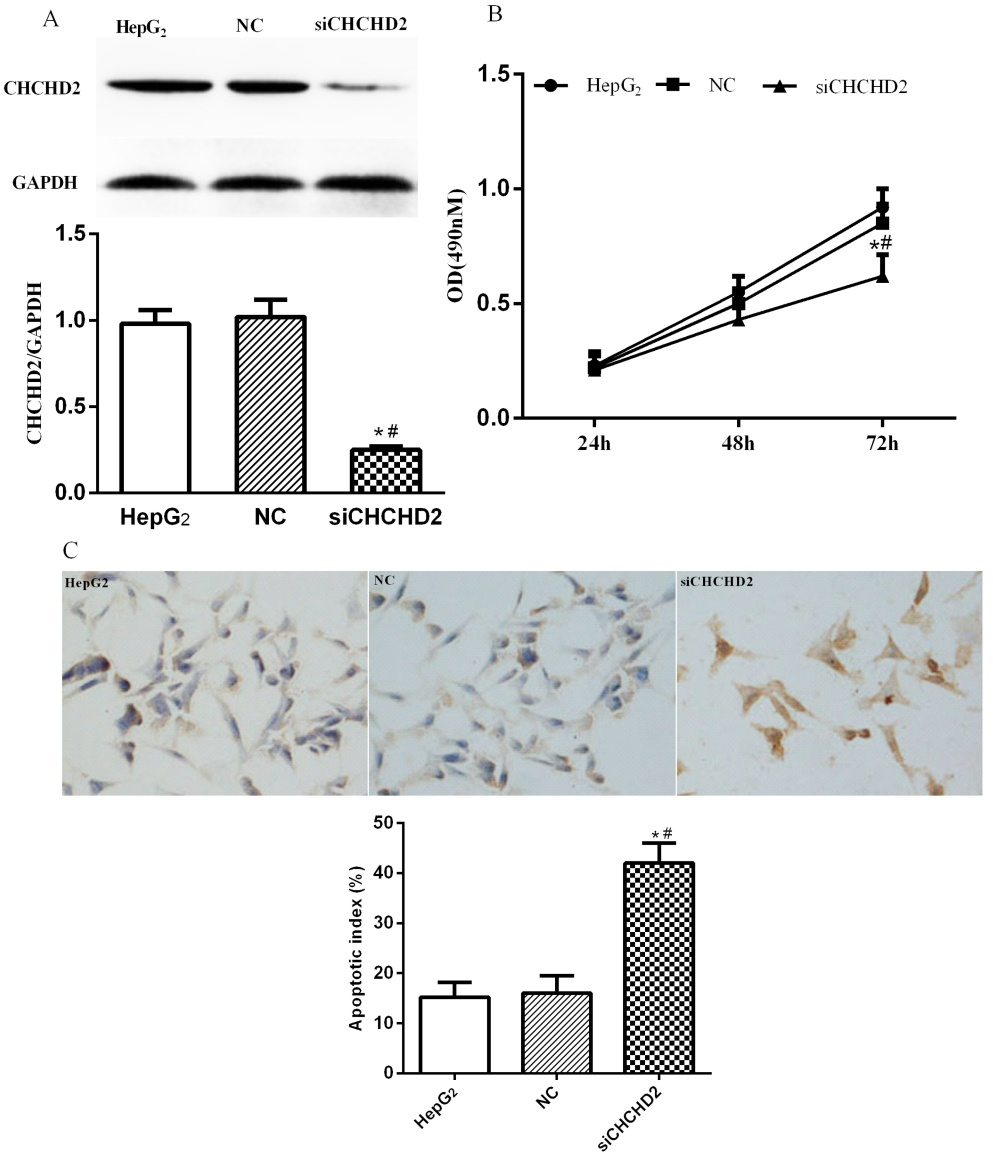
CHCHD2 knockdown decreased HepG_2_ cell proliferation and enhanced apoptosis. HepG_2_ cells were transfected with CHCHD2 specific siRNA or NC siRNA. (A) CHCHD2 protein level was determined by Western blot analysis. (B) The proliferation of HepG_2_ cells was measured by MTT assay. (C) The apoptosis of HepG_2_ cells was assessed with TUNEL assay. Data were presented as means±SD (n=3). ^*^ *P*<0.05, compared to the untreated group; ^#^ *P*<0.05, compared to NC group.

To investigate cellular function of CHCHD2 in HCC cells, we depleted it in HepG_2_ cells via siRNA-mediated knockdown (Fig. 3A). The knockdown of CHCHD2 led to significantly decreased proliferation in HepG_2_ cells (Fig. 3B). In addition, TUNEL assay indicated that the untreated cells and cells treated with control siRNA showed regular cell morphology and few light-colored cells, while HepG_2_ cells treated with CHCHD2 specific siRNA showed brownish yellow nuclei, chromatin condensation and irregular cell morphology, suggesting that CHCHD2 depleted cells had higher apoptotic index (Fig. 3C).

### CHCHD2 knockdown suppressed migration of HCC cells

**Figure 4:**
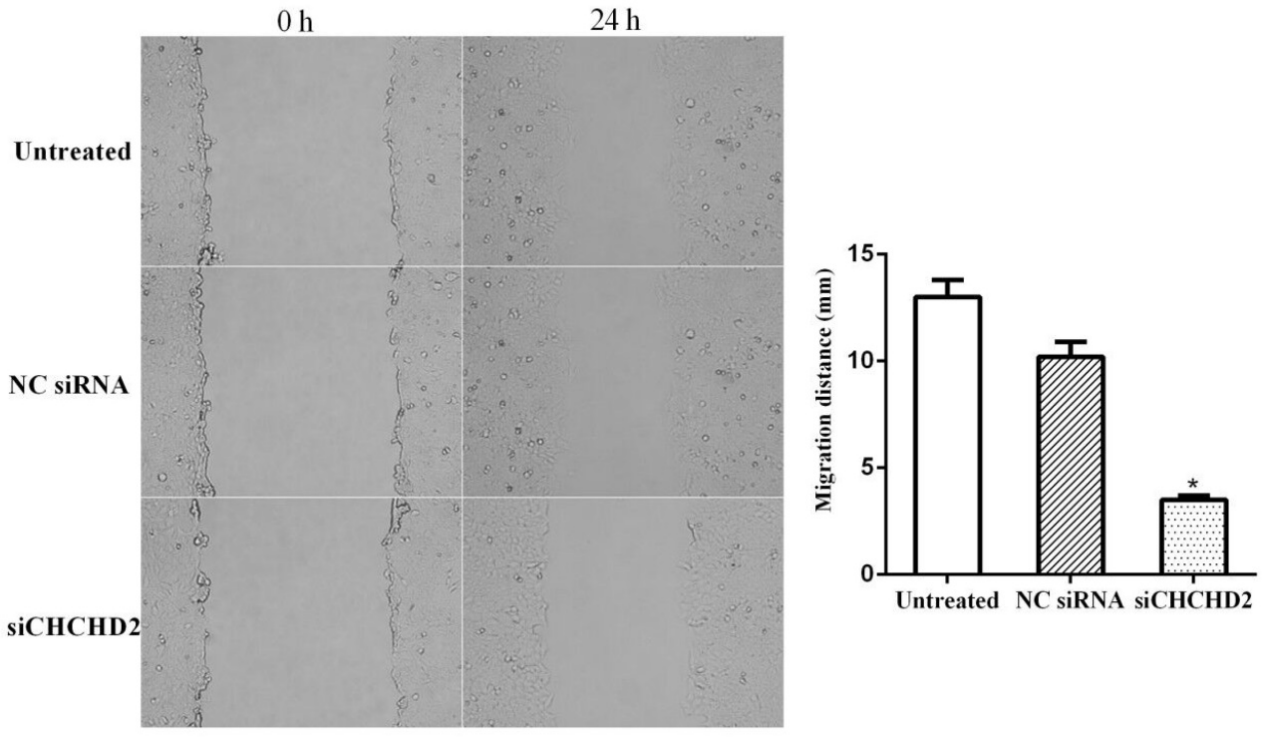
CHCHD2 knockdown suppressed HepG_2_ cell migration. HepG_2_ cells were transfected with CHCHD2 specific siRNA or NC siRNA, and a line was scratched with a 10 μl pipette tip. The filling of the space by cell migration was detected 24 h later. Data were presented as means±SD (n=3). ^*^ *P*<0.05, compared to untreated or NC siRNA treated cells.

Wound healing assay showed that the migration capacity was significantly diminished in HepG_2_ cells depleted of CHCHD2 compared to the untreated cells and cells treated with control siRNA, while there was no significant difference in cell migration ability between the untreated and cells treated with control siRNA (Fig. 4).

### CHCHD2 promoted angiogenesis of HCC

**Figure 5:**
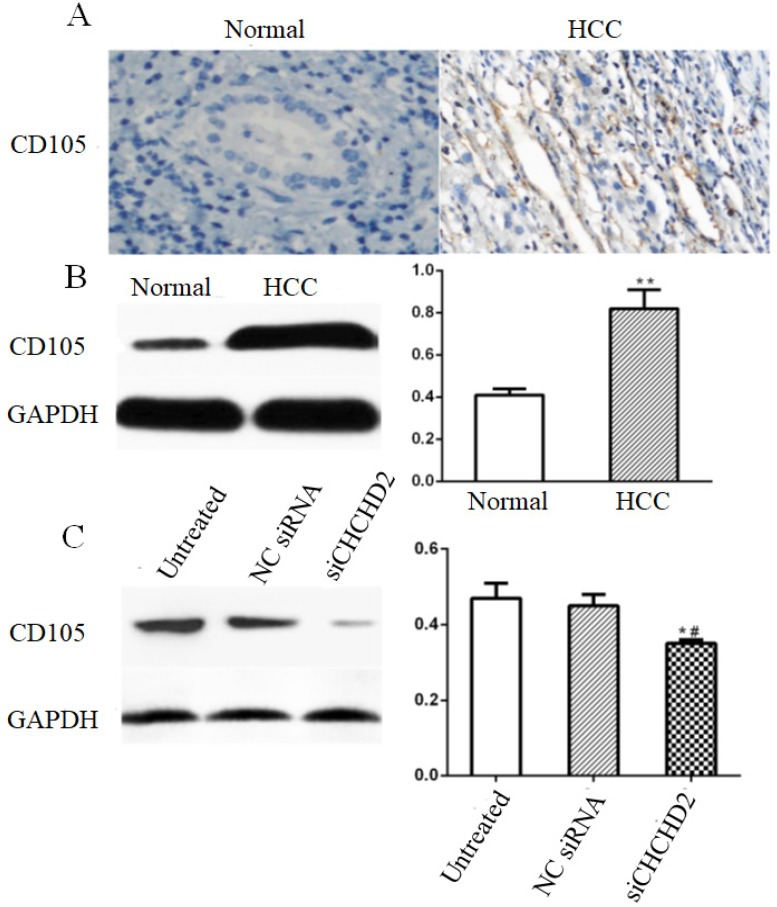
CHCHD2 enhanced angiogenesis in HCC. (A) Representative IHC images of CD105 expression in the array that contains HCC and normal liver tissues. (B) Western blot analysis of CD105 level in HCC tissues and surrounding normal tissues. Data were presented as means±SD (n=3). ^**^ *P*<0.01. (C) Western blot analysis of CD105 level in untreated, NC siRNA and siCHCHD2 transfected HepG_2_ cells. Data were presented as means±SD (n=3). ^*^ *P*<0.05, compared to untreated group; ^#^ *P*<0.05, compared to NC group.

Angiogenesis represents an important mechanism for cancer development and metastasis. CD105 is one of the most commonly used microvessel markers, which is only expressed in the endothelial cells of the tumor blood vessel 9. Immunohistochemical and Western blot analysis showed that HCC tissues had higher expression of CD105 compared to surrounding non-tumorous tissues (Fig. 5A and B). In addition, HepG_2_ cells depleted of CHCHD2 had lower CD105 level compared to the untreated cells and cells treated with control siRNA (Fig. 5C).

## Discussion

HCC is one of the most commonly diagnosed malignant tumors and represents the third leading cause of cancer-related death globally 10. The late diagnosis and high post-operative recurrence rate remain the major barriers against long-term survival of HCC patients 11. Therefore, it is urgently required to develop reliable prognostic biomarkers to predict the outcome of patients and identify novel therapeutic targets. In this study, we demonstrated that CHCHD2 was a potential biomarker for predicting disease progression and overall survival in patients with HCC and revealed that CHCHD2 was involved in cell proliferation, migration and angiogenesis in HCC.

In recent years, mutations of CHCHD2 and CHCHD10 are widely linked to a series of neurodegenerative diseases, including Parkinson's disease, amyotrophic lateral sclerosis and frontotemporal lobe dementia 12-14. However, the role of CHCHD2 in cancer remains unclear. Analysis of mRNA microarray expression data obtained from the Oncomine database shows that CHCHD2 is overexpressed in many types of cancers, including breast cancer, glioblastoma, leukemia, lung adenocarcinoma and lymphoma 15-19. Consistent with previous reports, we found that CHCHD2 was highly expressed in HCC tissues compared to adjacent non-tumorous tissues. In addition, we found that high CHCHD2 expression was associated with poor differentiation, lymph node metastasis and high TNM grade of HCC. Survival analysis showed that the overall survival of low CHCHD2 expression group was significantly longer than that of high CHCHD2 expression group. These results suggest that CHCHD2 is involved in the occurrence and development of HCC and could serve as a prognostic biomarker for HCC.

We then explored the mechanisms underlying potential oncogenic role of CHCHD2 in HCC. We found that silencing of CHCHD2 in HepG_2_ cells led to decreased cell viability and increased cell apoptosis. Liu *et al.* reported that CHCHD2 bound to Bcl-xL and inhibited the mitochondrial accumulation and oligomerization of Bax to negatively regulate mitochondria-mediated apoptosis 5. This mechanism may explain enhanced apoptosis in HepG_2_ cells depleted of CHCHD2. Cell migration represented an important aspect of cancer metastasis. We therefore assessed the migratory capacity of HepG_2_ cells depleted of CHCHD2 and the results showed a significant inhibition of migration of HepG_2_ cells after CHCHD2 knockdown. In agreement with our results, Wei *et al.* reported that cell migration was significantly decreased in CHCHD2 depleted non-small cell lung cancer (NSCLC) cells 6.

Tumor angiogenesis is a prerequisite for the growth of a variety of tumors including HCC 20. CD105 is a marker for tumor angiogenesis and is correlated to tumor microvessel density 9,21. We found that CHCHD2 and CD105 were both upregulated in HCC tissues compared to non-tumorous tissues. In addition, knockdown of CHCHD2 resulted in downregulation of CD105 expression. These data suggest that CHCHD2 may be involved in HCC angiogenesis. However, further in* vitro* and in* vivo* studies are needed to elucidate molecular mechanism underlying the role of CHCHD2 in tumor angiogenesis.

In summary, CHCHD2 is upregulated in HCC tissues and high CHCHD2 level is associated with poor differentiation, lymph node metastasis, local tissue invasion, high TNM grade and poor prognosis. Silencing the expression of CHCHD2 inhibits HCC cell proliferation and migration while induces apoptosis of HCC cells. These results suggest that CHCHD2 may play an oncogenic role in HCC and CHCHD2 is a potential biomarker for poor outcome of HCC patients.

## Figures and Tables

**Table 1:** Correlation of CHCHD2 expression to clinicopathological features of HCC patients

Clinicopathological features	No.	CHCHD2		*p*
		-	+	
**Gender**				
Male	86	24	62	0.775
Female	58	18	40	
**Age (years)**				
≥50	76	22	54	0.564
<50	68	24	44	
**Tumor size (cm)**				
≤3 cm	54	18	36	0.547
>3cm	90	24	66	
**Differentiation**				
Well/moderate	90	40	50	0.007**
Poor	54	8	46	
**Lymphatic invasion**				
Negative	66	22	44	0.005**
Positive	78	6	72	
**TNM stage**				
Stage I/II	62	22	40	0.039*
Stage III/IV	82	12	70	

HCC: hepatocellular carcinoma. Means of different groups were compared by χ^2^ test. * *p*<0.05, ** *p*<0.01.

## References

[1] Ferlay J, Colombet M, Soerjomataram I (2019). Estimating the global cancer incidence and mortality in 2018: GLOBOCAN sources and methods. Int J Cancer.

[2] Zeng H, Zheng R, Guo Y (2015). Cancer survival in China, 2003-2005: a population-based study. Int J Cancer.

[3] Ke Y, Bao T, Zhou Q (2017). Discs large homolog 5 decreases formation and function of invadopodia in human hepatocellular carcinoma via Girdin and Tks5. Int J Cancer.

[4] Ke Y, Bao T, Wu X (2017). Scutellarin suppresses migration and invasion of human hepatocellular carcinoma by inhibiting the STAT3/Girdin/Akt activity. Biochem Biophys Res Commun.

[5] Liu Y, Clegg HV, Leslie PL (2015). CHCHD2 inhibits apoptosis by interacting with bcl-x l to regulate bax activation. Cell Death Differ.

[6] Wei Y, Vellanki RN, Coyaud E (2015). CHCHD2 is coamplified with egfr in nsclc and regulates mitochondrial function and cell migration. Mol Cancer Res.

[7] Liu Y, Zhang Y (2015). CHCHD2 connects mitochondrial metabolism to apoptosis. Mol Cell Oncol.

[8] Li L, Wei Y, To C (2014). Integrated Omic analysis of lung cancer reveals metabolism proteome signatures with prognostic impact. Nat Commun.

[9] Cho T, Shiozawa E, Urushibara F (2017). The role of microvessel density, lymph node metastasis, and tumor size as prognostic factors of distant metastasis in colorectal cancer. Oncol Lett.

[10] Grandhi MS, Kim AK, Ronnekleiv-Kelly SM (2016). Hepatocellular carcinoma: From diagnosis to treatment. Surg Onc.

[11] Makramalla A, Itri JN, Choe KA (2016). Transarterial therapies for hepatocellular carcinoma. Semin Roentgenol.

[12] Liu Z, Guo J, Li K (2015). Mutation analysis of CHCHD2 gene in chinese familial parkinson's disease. Neurobiol Aging.

[13] Yang X, An R, Zhao Q (2016). Mutational analysisi of CHCHD2 in chinese patients with multiple system atrophy and amyotrophic lateral sclerosis. J Neurol Sci.

[14] Zhou ZD, Saw WT, Tan EK (2017). Mitochondrial CHCHD-containing proteins: physiologic functions and link with neurodegenerative diseases. Mol Neurobiol.

[15] Liang Y, Diehn M, Watson N (2005). Gene expression profiling reveals molecularly and clinically distinct subtypes of glioblastoma multiforme. Proc Natl Acad Sci USA.

[16] Yanai I, Benjamin H, Shmoish M (2005). Horn-Saban S, Safran M, Domany E, Lancet D, Shmueli O. Genome-wide midrange transcription profiles reveal expression level relationships in human tissue specification. Bioinformatics.

[17] Liu M, Zhang X (2017). An integrated analysis of mRNA-miRNA transcriptome data revealed hub regulatory networks in three genitourinary cancers. Biocell.

[18] Andersson A, Ritz C, Lindgren D (2007). Microarray-based classification of a consecutive series of 121 childhood acute leukemias: Prediction of leukemic and genetic subtype as well as of minimal residual disease status. Leukemia.

[19] Landi MT, Dracheva T, Rotunno M (2008). Gene expression signature of cigarette smoking and its role in lung adenocarcinoma development and survival. PloS One.

[20] El-Emshaty HM, Saad EA, Gouida MS (2018). Associations between CD133, CK19 and G2/M in cirrhotic HCV (genotype-4) patients with or without accompanying tumor. Biocell.

[21] Goldiş DS, Sferdian MF, Tarţă C (2015). Comparative analysis of microvessel density quantified through the immunohistochemistry expression of CD34 and CD105 in rectal cancer. Rom J Morphol Embryol.

